# The patient experience in community mental health services for older people: a concept mapping approach to support the development of a new quality measure

**DOI:** 10.1186/s12913-018-3231-6

**Published:** 2018-06-18

**Authors:** Mark Wilberforce, Eric Batten, David Challis, Linda Davies, Michael P. Kelly, Chris Roberts

**Affiliations:** 1 Social Policy Research Unit, Department of Social Policy and Social Work, University of York, York, UK; 2 EDUCATE group, Stockport, UK; 3 PSSRU, School of Health Sciences, University of Manchester, Manchester, UK; 4 Centre for Health Economics, School of Health Sciences, University of Manchester, Manchester, UK; 5 Primary Care Unit Institute of Public Health, University of Cambridge, Cambridge, UK; 6 Centre for Biostatistics, School of Health Sciences, University of Manchester, Cambridge, UK

**Keywords:** Patient experience, Dementia, Concept mapping, Community mental health, Social care

## Abstract

**Background:**

The patient experience is a crucial part of the measurement of service quality. However, instruments to evaluate experiential quality in the community mental health care of older adults are lacking. Before designing a new instrument, clarity is needed about what is to be measured, and how care experiences are articulated by patients. The study aimed to construct a framework to describe older patients’ experience of community mental health and social care.

**Methods:**

Concept mapping blends structured qualitative data collection with quantitative analysis in a mixed method approach. Five activities were undertaken. Patients first identified sentences describing the care experience; a card-sort exercise then grouped these thematically; multidimensional analysis portrayed these data in a map of clusters; interpretation was by patient advisers; finally a new questionnaire was designed. The research involved 22 older people with mental health problems and 29 mental health practitioners, from one region of England.

**Results:**

Sixty-seven statements were identified that described the care experience. Analysis of card sort data revealed seven clusters, which were interpreted by patient advisers to the study as: personal qualities and relationships; communication problems; feeling powerless; in-and-out care; bureaucracy; focus on life, not just mental health; and continuity of care. These themes and the component statements were used as a foundation for later work, developing a new measure of the care experience in mental health services for older people.

**Conclusions:**

Concept mapping has many strengths as an empirical and participant-driven means for underpinning new measurement instruments. A group of older people identified 67 candidate statements that could act as questionnaire items grouped within seven themes. Future research will establish the psychometric properties of the new measure.

**Electronic supplementary material:**

The online version of this article (10.1186/s12913-018-3231-6) contains supplementary material, which is available to authorized users.

## Background

Mental health problems are a significant cause of distress to many older people and their families. An estimated 47 million people live with dementia worldwide, with this figure predicted to double every two decades [1]. Depression affects an estimated 20% of people aged over 65, whilst older adults may additionally experience the full spectrum of other mental health problems [2]. The consequences can be severe, with daily functioning, social relationships, and psychological wellbeing all at risk of deterioration.

Central to meeting the needs of this group are specialist community support services. In England, people with complex care needs unable to be managed within primary care may be referred for specialist support through a community mental health team (CMHT). CMHTs are an internationally recognised model of secondary service provision [3] comprising a mix of professional disciplines spanning psychiatry, mental health nursing, occupational therapy, social work and psychology [4]. Importantly, CMHTs are able to directly access a range of personal and social care services, including specialist community care services for older people with dementia and other support needs. These are commonly delivered by separate organisations under contract to local commissioners, but CMHT practitioners may remain responsible for their coordination and reviewing successful delivery [5].

As in any other health or care service, improving quality is of central concern for all stakeholders. In England, the National Health Service defines quality as comprising three attributes: effectiveness, safety, and patient experience [6]. What constitutes the patient experience is contested, but for the present, it can be understood to be a personal evaluation of the interactions with health services in the context of arranging or receiving care [7]. Research using stated preference techniques indicates that experiential features of care are highly valued by patients [8], and that this may be particularly true for older adults using community mental health services [9]. The experience of receiving care can have a profound impact on the wellbeing of older adults with mental health needs. Positive examples include care visits helping to address social isolation and reinforcing personal identity [10–15] and contributing to self-worth through valued interpersonal relationships [9, 14, 16–19]. Yet negative examples are also common within the literature with standardized and depersonalized community services contributing to a “damaging sense of personal erasure” [20].

For quality improvement and scientific study, the accurate and efficient measurement of care experiences is crucial. Yet in community mental health care, experience measures arguably use a vocabulary that attends more to the priorities of working-age adults. It has consistently been argued that interpretations of patient experience have a “youthful bias” [21] with a focus on individuality, recovery, independence and autonomy in care, despite evidence that priorities may have different emphases amongst older adult groups with complex needs [22, 23]. Further, satisfaction metrics and related instruments (e.g. the Friends and Family Test), with roots in consumerist approaches to public service delivery, are routinely used as proxies for patient experience despite profound doubts over their validity when used with older people [24]. A systematic review of quality measures relevant to older people’s services and person-centred care identified substantial gaps in their breadth and rigour [25]. The authors thus set about establishing a new instrument for use in community mental health services.

In the development of such measures, researchers must first attend to concept elicitation, which defines the ‘evaluative space’ [26] that an instrument will address, and its constituent parts. Despite being a self-evidently important stage in instrument design, it has paradoxically received least attention in methods development and has been described as lacking transparency [27]. There is no agreement on an optimal approach, although it is widely expected that patient-reported measures should be developed from the voices of patients themselves, to avoid researcher and clinician pre-suppositions [28].

This new study aimed to construct a conceptual framework for describing the patient experience of using community mental health services, and how it would contribute to the subsequent development and testing of a new patient experience measure.

## Method

One method for concept elicitation gaining traction in the measure development literature is concept mapping [29]. Concept mapping is a multi-stage and mixed-method approach for exploring how individuals perceive a phenomenon of interest. It comprises five separate research activities, spanning both qualitative and quantitative formats of data collection. Stages engaging patients were first piloted and refined with four patients/carers receiving support from community mental health services, who acted as advisers to the study.

### Stage 1: Item generation

Concept mapping begins with a group of participants brainstorming statements that describe the phenomenon of interest up to a point of saturation, where no further ideas are forthcoming. To this end, a face-to-face meeting was convened with a sample of older adults and family carers recruited from a community mental health support service in North West England. The service provided 1:1 and group-based support with social care needs overseen by a care coordinator from the community mental health team. Participants were invited by care workers ensuring people with both memory and mood difficulties were represented, although only individuals with capacity to consent were invited to join the study*.*

The meeting began with a scene-setting introduction by the lead author (MW), using visual materials describing mental health and care that spanned a range of health and social support, including assistance with activities of daily living, assessment tasks, medical treatment, emotional support, giving information and advice, arranging or delivering social care, and supporting informal carers. The focus question was then presented: “*what words and sentences describe a good care experience*?” During the subsequent discussion, the researcher wrote down the generated sentences on a wall display but did not edit them, permitting participants to control the precise wording. After completion, the list was reviewed by the authors and patient advisers against the focus question to ensure relevance.

### Stage 2: Card sort and rating

The second stage was a card sorting exercise. This was undertaken at a separate meeting, shortly after the first. However, the initial sample fell short of the number required to power subsequent analysis [30]. Consequently, the sample was extended to include practitioners in four local CMHTs. Participants were handed A5 cards with all the statements printed (one per card), and each was asked to sort the cards into piles “in a way that makes sense to you” [29]. The only instructions bounding the exercise were that each card must be sorted, even if that was into a pile on its own; and that a card could only be in one pile. Participants were finally asked to rate each item using a five-point Likert scale evaluating how important the statement was in care quality.

### Stage 3: Representation of statements

The card sort data were entered in matrix form (the ‘sort matrix’) showing, for each pair of statements, the total number of times across the sample that the items were sorted into the same pile. Non-metric multidimensional scaling (MDS) of this matrix was conducted in Stata (see Additional file 1). MDS helps to identify structure by representing items in two-dimensional space, (the ‘point map’) with distances between items representing their (dis)similarity. Where clusters of items are positioned together, these are considered to potentially represent a broader theme. The rigor of the proposed solution was tested through the Kruskal stress statistic [31], and Pearson’s Product-Moment correlation between the original sort matrix and the fitted distances (the ‘distance matrix’). Sensitivity analyses examined the implications for the MDS results of omitting statements that appeared conceptually difficult to interpret, and of pooling together service user and practitioner card sort data.

To assist subsequent interpretation of the point maps, a hierarchical cluster analysis was undertaken. Following Trochim’s classic text [29], the X-Y coordinates arising from the MDS analysis were clustered using Ward’s algorithm. This has the effect of partitioning the MDS solution into mutually exclusive clusters. Results were presented visually in a dendrogram.

### Stage 4: Interpretation

The results of the MDS and cluster analyses were first reviewed by all authors to inspect the quality of fit and assign a preliminary categorization of clusters. This was achieved by reviewing the point map and dendrogram to form a provisional ‘cluster map’ (the original point map with potential boundaries superimposed). Some researcher judgement was employed at this stage. Specifically, the researchers were not satisfied with the MDS positioning and subsequent cluster analysis in respect of nine of the statements. This is fully described within the findings.

However, the researchers made no attempt at a narrative interpretation of the clusters at this stage. An interpretation meeting was held separately involving the four patient/carers advising the study. They were then invited to review each cluster and consider (i) whether the items reflected a feature of care quality that resonated with their experience; and (ii) how to define and label the theme it represented. The meeting was audio-recorded, transcribed, and narratively summarized (supported by verbatim quotations) under the headings for each cluster.

### Stage 5: Utilisation

The final stage of concept mapping places the results into practical use, to meet the ultimate objectives of the exercise. In this instance, the concept map and underpinning statements formed the foundations of a new multi-item measure of service quality. It is not the purpose of this article to provide a full account of this development work, which will be the subject of separate publication. However, a brief narrative description is provided of how the concept mapping findings would be adapted to form a questionnaire for further psychometric testing.

The research was approved by an NRES (National Research Ethics Service) ethics committee (Ref: 14/NW/0303) and completed in February 2015. Informed consent was obtained from all individual participants included in the study.

## Results

### Item generation, sorting and rating

Thirteen patients and carers participated in the item generation stage; their characteristics are described in Table 1. The group identified 74 initial statements describing the care experience. Seven statements were removed since they could not be used to describe the quality of the care experience. Items are shown in Table 2. Card sorting was undertaken by fourteen patients and carers (including five new participants due to attrition between meetings) and 29 practitioners. On average, the 43 card sort participants created eight piles of cards with eight items in each. The most refined card sort comprised 17 piles, whilst the least refined grouped all cards into only four piles.

**Table 1:** Participant characteristics at the item generation stage

		n
Respondent	*Patient*	10
	*Carer*	3
Gender	*Female*	8
	*Male*	5
Age	*< 79*	7
	*> 80*	6
Living arrangements	*Lives alone*	8
	*Lives with other*	5
Ethnicity	*White British*	12
	*White Other*	1
Main mental health problem	*Organic*	5
	*Functional*	8
Service experience	*Specialist community mental health support*	13
	*Home support*	11
	*Day support*	9
Total participants		13

**Table 2:** Items arranged by cluster

*Cluster A: Personal qualities and relationships.* 18. I appreciate the “personal touch” 13. I appreciate the human contact 12. A smile and a warm handshake is important 15. It’s important that mental health care workers are gentle and tender 14. I like it when mental health care workers can sit, talk and take time to just be with me 27. I like it when they just sit with me sometimes 19. Mental health care workers should be good, honest people; showing kindness and consideration 58. It’s important that I am treated with compassion, like I matter 69. People should acknowledge me and respect me. 62. My care worker really listens to me; not just to what I say, but what it all means to me 33. I like mental health care workers to “share” as well as “care” 68. It’s important that they show an interest in me, beyond just the tasks and paperwork at hand 11. Good support leaves me feeling safe and secure 46. I need to feel secure and have mental health care workers give me reassurance 66. I appreciate mental health care workers getting to know me, by visiting me regularly 67. I value care-rs, not just do-ers. I miss the “care” part. *Cluster B: Communication problems.* 1. I want mental health care workers to talk to me, not at me. 32. Sometimes it takes me a bit of time to say what I mean 2. It’s important that people listen to what I am saying. 16. I want someone to speak-up for me 10. I feel uncomfortable if mental health care workers don’t speak and understand my language 31. I expect what I say to be taken seriously 73. Some mental health care workers use jargon I don’t understand 60. When they tell me off for doing things myself, I feel like a child 72. Sometimes they don’t look up from the notes when talking to me 36. I get frustrated when mental health care workers don’t pay attention to what I am trying to say 4. I shouldn’t have to speak-up and say “you’re not listening to me” 17. They should explain things clearly to me, and help me to navigate the system 37. I feel belittled when they try to persuade me that my problems are different to what I think they are 49. I feel that I shouldn’t have to complain. They should listen more carefully 3. If I need to complain about my care, they shouldn’t presume it’s because I have mental health problems 50. I’m worried that they will label me as ‘argumentative’ if I complain to them	*Cluster C: Feeling powerless* 47. I shouldn’t have to ring mental health care workers to chase-up what is going on 56. It is annoying when different professionals don’t to talk to each other 45. I shouldn’t have to be correcting them, and telling them what they need to be doing 38. Sometimes people on the phone are obstructive; they fob me off *Cluster D: In-and-out care.* 9. When they are rushed they can’t give you their full attention 25. I sometimes feel like I’m in the way because they’ve got so much to do 35. I don’t feel valued when they are just “in and out” 52. They don’t have time for me nowadays 24. Having to wait for mental health care workers makes me feel unwanted 53. I get frustrated having to sit and wait for the transport in the lounge 34. They should come at a time that suits me 6. Mental health care workers should be on time *Cluster E: Bureaucracy.* 41. Mental health care workers shouldn’t be stopped from doing their job by rules, bureaucracy and “health and safety” 5. Services should be available in all areas, not centralised in one area 7. I should have a say in what services are available locally *Cluster F: Focus on life not just mental health* 51. I value flexible support; for them to help me with life’s little things that matter to me 23. I like meetings to be interactive; doing things together rather than them doing it all 26. It’s important they support me to do activities that matter 55. The choice of different activities my mental health care workers involve me in is excellent 28. Good support is flexible so I can have variety in the activities that I’m supported to do 29. I like mental health support to keep me involved and busy 74. Some variety would be nice, so that I can be supported to cook 63. My mental health care workers keep me connected and involved with my community 71. Good mental health care workers are an important part of the community 30. Good support brings people together and keeps me in touch *Other items: Continuity of care from people who get to know you* 54. There’s a lack of continuity, so you can’t get to know people. 40. I expect new mental health care workers to read my notes before they come. 39. It’s embarrassing to be washed and dressed by a stranger. 57. Some mental health care workers are so fixed in their ways. They just do they job written on their paperwork. 64. I should be asked about what goes into my support plan. 42. They should recognise that if they see me on a “good day”, next time might be a “bad day”. 44. It’s frightening when you don’t know who it is coming to see you this time 59. Care has to be at a pace that suits me. There’s no point rushing me. 70. I shouldn’t be left in an unsafe situation, like cooking for myself.

**Fig. 1:**
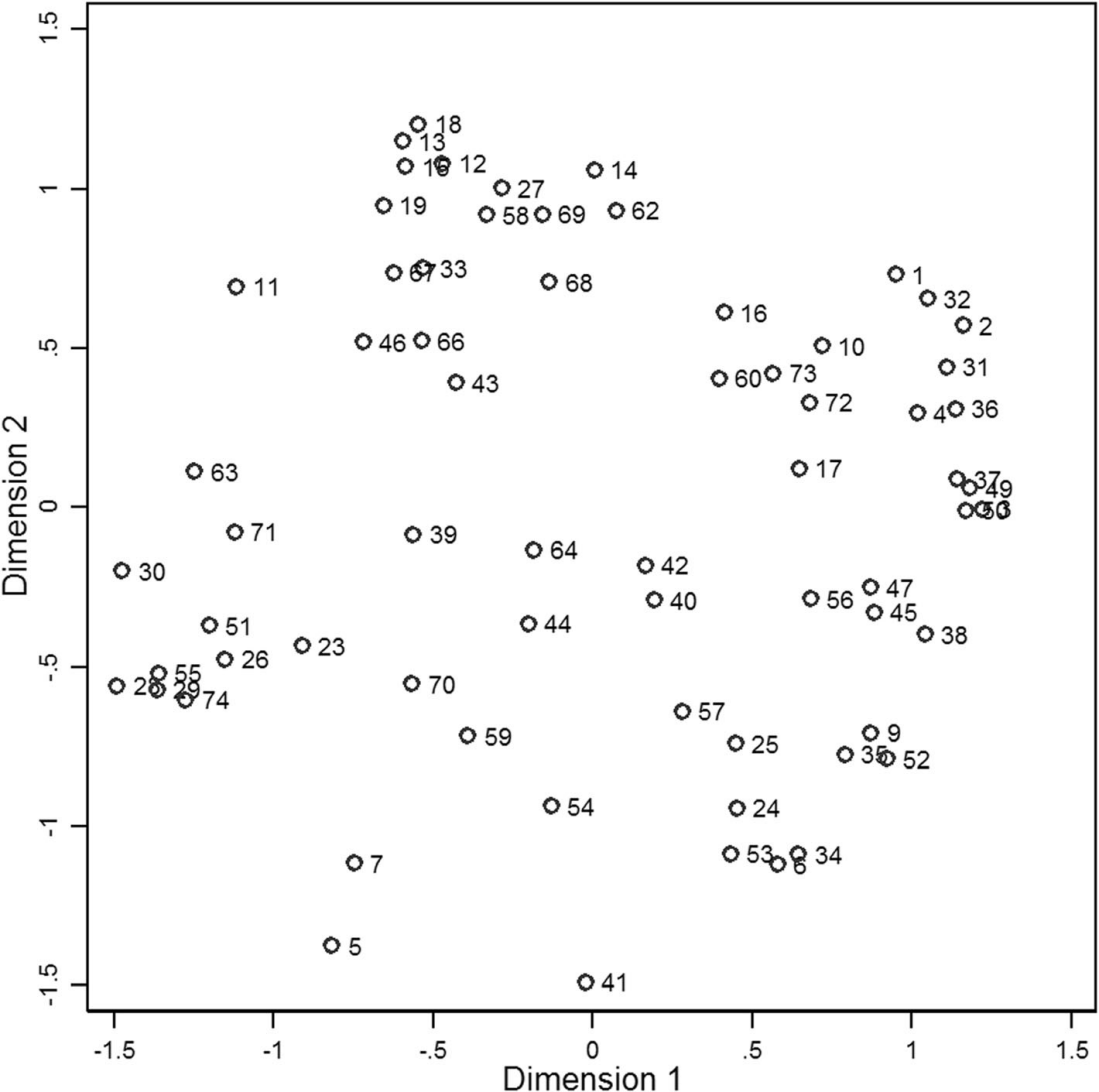
Point map from multidimensional scaling

MDS analysis was performed on the card sort data resulting in the point map presented in Fig. 1. The solution achieved a Kruskal stress score of 0.203. The correlation between the original sort matrix and fitted distance matrix was 0.878, suggesting 77% shared variance between the input and output data. The dendrogram from the accompanying cluster analysis is presented as Additional file 2.

**Fig. 2:**
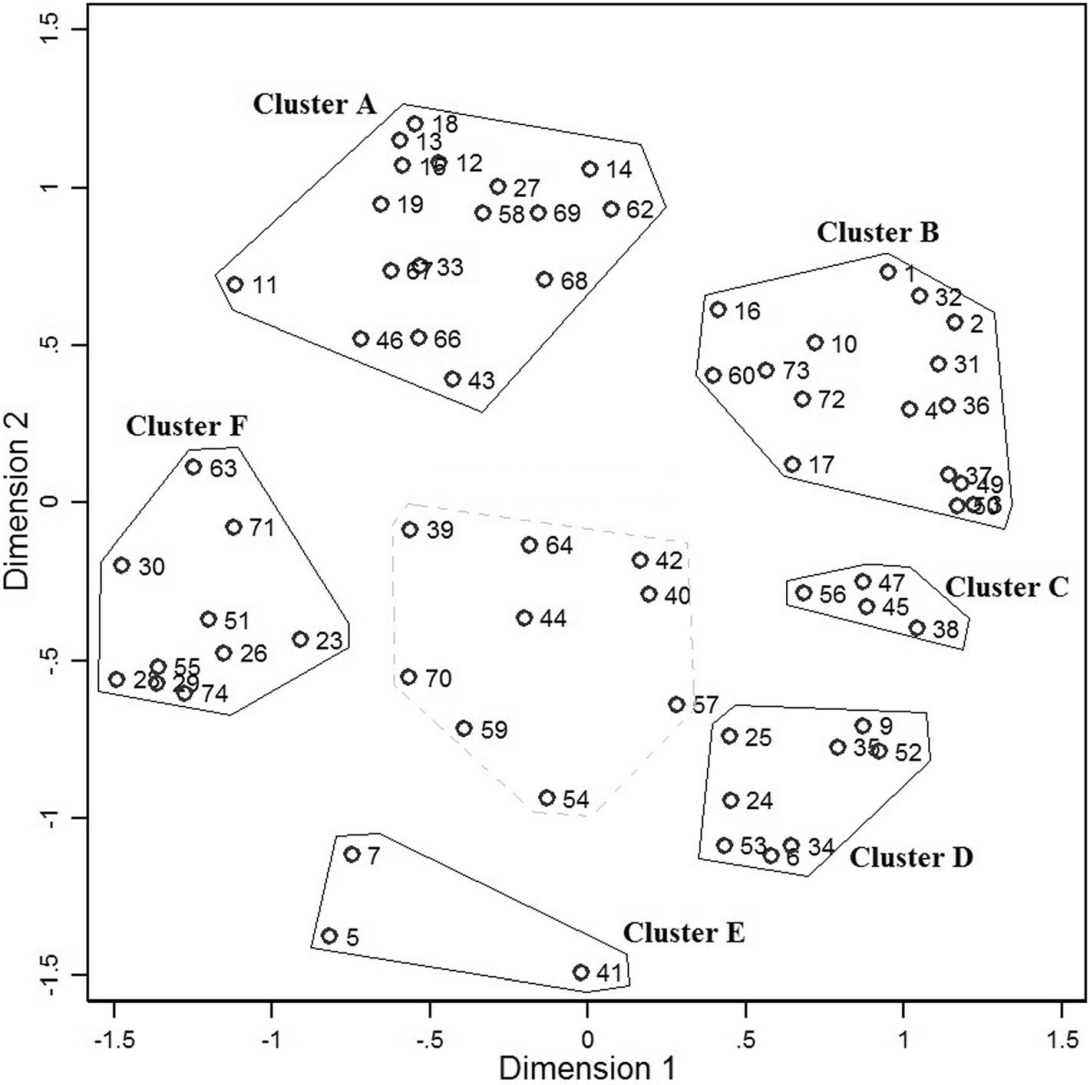
Cluster map from multidimensional scaling and preliminary interpretation

The authors made a preliminary identification of six clusters shown in Fig. 2. The items within each cluster appeared to relate to similar themes, whilst being *dis*similar to items in other clusters. However, two issues required resolution.

First, nine items located in the centre of the map were difficult to cluster in a meaningful way. Their content appeared to be poorly suited to the clusters identified by the dendrogram, but also did not seem easy to fit into other clusters. The mean correlation between the original sort and fitted distance matrices for these nine items was 0.620, which was noticeably lower than for all items combined (*r* = 0.878). As an additional sensitivity test, these items were removed and the MDS re-estimated, with the resulting solution achieving a superior Kruskall stress score of 0.171. Removing these items had no important consequences for the clustering of other items. Potentially, these were idiosyncratic items, or poorly phrased, which caused participants to sort them in an inconsistent manner. This interpretation was supported by inspecting the raw sorting pattern for these items, which revealed a very wide distribution of sorting frequencies *vis-à-vis* other items, with only items 39 and 44 being commonly sorted together. A second difficulty arose as to whether Clusters B and C were sufficiently distinct, or should be combined into a single theme (as recommended by the dendrogram). It was decided, for both these issues, to seek the advice of the patient advisers at the interpretation stage.

Additionally, a sensitivity analysis tested the implications of merging the service user and practitioner card sorts. MDS analysis was conducted for each sample. The results indicated that the same clusters could be clearly identified in both samples. The correlation between the distance matrices for the two samples was reassuringly high (*r* = 0.860). However, three items (9, 57 and 62) in the service user point map were located outside of the cluster assigned in the aggregate analysis, indicating that these items may be interpreted differently by service users than by practitioners.

Finally, importance ratings confirmed that the items were generally regarded as valuable for the patient experience in this sample (Table 3). There was some tentative indication that factors relating to interpersonal qualities and communication skills were more highly regarded than the timing and organisation of care, but estimates had wide confidence intervals.

**Table 3:** Important rating for each cluster

Cluster	Mean	Standard deviation
A: Personal qualities and relationships	4.23	0.565
B: Communication problems.	4.33	0.574
C: Feeling powerless	4.19	0.774
D: In-and-out care	3.89	0.719
E: Bureaucracy	3.74	0.927
F: Focus on life not just mental health	4.06	0.765
Other: Continuity of care	4.09	0.638

### Interpretation

#### Cluster a: Personal qualities and relationships

The patient advisers considered that the items in Cluster A formed a coherent theme and one that was important to the care experience. They perceived that the theme related to personal qualities and care relationships. The group noted a number of “warm adjectives” referring to mental health care workers, and one member summarised them as meaning that care incorporated “a sense of humanity”. When prompted on what this meant, she responded that mental health care workers can easily forget “to do the things we all should practice in our daily lives towards other people”. To this member, it meant “just demonstrating that you’re a real person” and not providing support mechanically. Item 62 (*My care worker really listens to me; not just to what I say, but what it all means to me*) resonated particularly well with the group, who stressed that problems should be set in wider life context. One said that it meant that they are “join[ing] in with us, not just listen[ing] and moving on”. The group also pointed to the reassurance that good care workers can bring, and that this was part of an understanding and empathetic approach to care. It was also noted that the items illustrated the different ways that caring relationships can be expressed, from careful listening and understanding, to tactile responses represented by items 18 (*I appreciate the ‘personal touch’*) and 12 (*A smile and a warm handshake is important*).

#### Cluster B (communication problems) and cluster C (feeling powerless)

The group discussed the items in Clusters B and C, and an initial reaction was that they collectively represented difficulties in communicating and poor listening skills on the part of mental health care workers. One member of the group felt that these items captured his own experience well. He gave an example of a mental health nurse spending an appointment continually looking at her notes, not making eye contact or showing evidence of active listening. In his example, he felt that he did not have an opportunity to make sure that the care worker had understood what he had said, and vice versa, since he felt that the meeting was regimented around an agenda he had not seen. Others agreed, saying that this would lead to only a superficial understanding of problems that were more complex, or would prevent an appreciation of concerns or fears that existed beneath the surface. The group felt that some items reflected service users “having a moan; a complain”, but recognised this as the consequences that poor communication could have on the care experience.

The group’s attention then turned to a subset of four items (47, 56, 45, 38) that were identified in the cluster map as belonging to a different group (Cluster C) to the others. One member began this discussion by saying they *were* different to the others in Cluster B, saying “it’s not just [the worker is] not listening… they’re doing something else as well”. When prompted for further elaboration, she reflected that these items implied some “obstruction” being put in place that “wilfully” made it difficult for views to be acted upon. The tenor of these items was also identified as different, with a “real angst” being expressed as if “they were coming up against a brick wall”, and “reaching the end of [their] tether”. The group identified with the emotive language being used, with one saying that: “we have a certain fellow-feeling with some of these people”.

#### Cluster D: In-and-out care

Participants also recognised many of the frustrations advanced in the eight items forming Cluster D. One member identified with item 25 (*I sometimes feel like I’m in the way because they’ve got so much to do*) saying that she had felt “deferential” because she “didn’t want them [mental health care workers] to be inconvenienced, and sometimes you feel a bit overcome when everything happens so fast”. Referring to home care support, another member of the group explained that her husband had been hurriedly awoken and bathed/dressed having been told “we’ve only got 10 minutes”. In that instance the care workers “were terribly contrite”, but she remarked that it left her husband feeling dazed. For some with limited social contacts, it was considered that a visit from a care worker might be the only human exchange for some time. However, the group felt some sympathy for mental health care workers under pressure to care for increasing numbers of service users. Of item 6 (*Mental health care workers should be on time*), one said: “that’s utopia!”.

#### Cluster E: Bureaucracy

This cluster comprised only three items, and thus relatively little conversation was sparked. However, the group latched onto the words ‘health and safety’ and ‘bureaucracy’, with one member referring to these as “those dirty little words”. She went on to talk about regular reorganisations within health and care providers causing confusion over who was responsible for delays in decisions about care being taken.

#### Cluster F: Focus on life not just mental health

These ten items prompted discussion about the purpose of mental health support for older adults, and included some impassioned contributions. Items 29 (*I like mental health support to keep me involved and busy*) and 26 (*It’s important they support me to do activities that matter*) were singled out as summarising group members’ views about the importance of “being occupied”. One spoke of a dementia café they attended and how - at the start – “one look around the room would see 15 people drowning”. She continued: “our world was folding in on ourselves; but now look at us”, reflecting on their improved position since. Another member interrupted:

“Yes – there is life after Alzheimer’s! [Thumps table]. There are so many people in [name of town] who I know with Alzheimer’s, but they never come out. Their world shrinks… That’s why I ride my bicycle. It’s not so much going somewhere, it’s a statement. It lifts me. Here I am!”*.*

#### Other items: Continuity of care from people who get to know you

Finally, the group discussed other items that were part of the card-sort, but which were not part of the cluster map, to see if they expressed features of the care experience not already discussed. One member perceived that item 39 *(it’s embarrassing to be washed and dressed by a stranger*) reflected an important idea about how social relationships in general can be “uncomfortable at first”. Relating to item 44 (*it’s frightening when you don’t know who it is coming to see you this time*)*,* the group member did not identify with the adjective used in this item, saying “it’s frightening? No, but it’s unnerving”.

However, the items did prompt members to reflect on the importance of continuity in care, which was an issue not sufficiently addressed in other clusters. The group reported that service users appreciated seeing a familiar face when being supported, saying: “they get to know what makes us tick” and “can see when we are struggling”. They also noted that it can be unsettling to have strangers come, who have not heard what has been already said to others.

Finally, the group reflected on the appropriateness of the labels assigned to each cluster. The group concluded that the seven key features of the care experience could be summarized as: *personal qualities and relationships; communication problems; feeling powerless; in-and-out care; bureaucracy; focus on life, not just mental health; and continuity of care.*

### Utilisation

The above stages provided a valuable source of items and a structure for a new instrument to evaluate the experiences of older people receiving long-term community support. Subsequent work (subject to separate publication) would go on to review the statements for potential use in a questionnaire arising from the study. The authors and patient advisers would scrutinise and re-phrase items so that respondents could use them to evaluate their care in line with their own experiences. Some amendments would be necessary to fit good practice in measurement design (e.g. removing double-negatives, dividing statements that address multiple features of care). Given that the concept map included a blend of both positive and negative statements, this would also be considered at the questionnaire development stage. Care would be taken to retain the specific terms used by concept mapping participants where possible. By remaining ‘true’ to participants’ voices the schedule could retain content validity and use a phrasing most appropriate to the population in which the questionnaire would be used. How well this was achieved would be explored in ‘cognitive testing’; an in-depth qualitative method used to examine how respondents process and interpret questions, and how they use their experiences to formulate an answer [32].

Future stages of the research will then psychometrically test the questionnaire in a large quantitative sample. That analysis will attend to aspects of measurement outside of the scope of concept mapping and its development stages, including an assessment of factor structure, test-retest reliability and criterion-related validity.

## Discussion

The study aimed to identity the key components of the patient experience in older adult community mental health services, as articulated by a sample of service users and carers. This was to serve as a basis for designing a new patient experience measure. To this end, concept mapping was chosen as an appropriate method. Its benefits include the combination of quantitative and qualitative methods, offering a richness of data and objectivity of statistical analysis. Furthermore, the method engages patients in a participative endeavor beyond their simply being a source of data. Through additional involvement in thematising items and interpreting results, patients’ influence on the research is enhanced.

### The validity of identified attributes

The group identified seven clusters of quality attributes. As a platform for a new measure, they can be regarded as credible, enduring themes of patient experience in older adult community mental health care. For example, the importance of care relationships to wellbeing and identity in later life has been long-recognised, especially amongst populations where social networks can be restricted [14]. Other research has emphasized that good quality home support must focus not only on the completion of caring tasks, but also on affective and inter-personal dimensions of support [14, 15, 33, 34]. The concept mapping identified several examples of the sorts of standards expected in communication, but the negative phrasing of these indicated that they are often forgotten. Many of the statements parallel directly Tom Kitwood’s [35] “malignant social psychology” in dementia care, such as the notion of being spoken ‘at’ or ‘over’, not ‘to’, and mirror repeated evidence that care workers are perceived not to communicate well with many older adults receiving care [36–39].

The items also address the long-standing issue of how older adults prefer to be engaged in care decision-making. It has been asserted that older adults do not value involvement in decision-making to the same degree as younger adults [40, 41], but this appears inconsistent with frustrations that older adults describe when their views and opinions go unheard [42]. More likely, it is that they prefer a less directive role in decision-making: being satisfied with being consulted and listened-to carefully, rather than directly controlling choices [22, 43].

The study also identified that care experiences were enhanced wherever it was in tandem with a positive focus on what the patient could still achieve and participate in the wider community, rather than a narrow focus on symptoms alone. Social isolation is a predictor of cognitive decline and mental distress in old age [44], and ameliorating such risks is a recognised goal for community mental health services [16]. A burgeoning body of research describe mechanisms for providing social activity for care home residents with dementia [45–48], but for people with mental health problems living at home there may be cause for concern with fewer such opportunities. Despite a long-standing commitment to expand community services to prevent more costly institutional care, UK and wider European macroeconomic austerity has impinged upon the ability of services to match demands [49].

### The value of concept mapping to questionnaire design

The first stage of any attempt to design a new instrument is to clarify the concept under measurement [50]. What are its component parts and how are these defined? The construction of a conceptual framework, design of questions and their implementation in a schedule is a complex process involving key decisions that shape the content validity of the measure [51]. Commonly, this stage receives far less attention than subsequent psychometric analysis. Concept mapping provides a systematic but flexible method to achieve these ends.

Researchers must weigh-up the advantages of concept mapping against the merits of other approaches [50]. Purely qualitative methods can be used to identify themes, and transcripts can provide illustrations of how these could be captured within a questionnaire item [52]. However such analysis unavoidably relies on researcher interpretation, and the in-depth nature of qualitative inquiry may risk identifying nuanced issues that would be outside the scope of structured questionnaires to evaluate. Arguably concept mapping can overcome these limitations, by engaging participants in theme formation (through card sorting) and interpretation, as well as using simple statements as the unit of analysis. However, in the absence of experimental data comparing the two approaches to conceptualisation and questionnaire development, such choices are left to researcher judgement.

### Limitations

The findings of the group concept mapping must be set in the context of the study’s limitations. First, the number of statements generated was fewer than some online concept mapping exercises [30], perhaps due to a relatively small number of participants (below the 15–20 recommended [53]). Nevertheless, a criticism of concept mapping is that where larger sets of statements have been created, researchers have then been forced to prune them to a manageable set for card sorting [54]. Therefore it may be preferable to have fewer, higher quality, statements in the first place. Second, the number of participants needed to be bolstered for card-sorting, which demands larger numbers to reach stable MDS solutions [30]. However, additional recruitment was of practitioners rather than other service users, primarily because of time constraints. Although this is common practice [54], it is not ideal. That said, the present study included a sensitivity analysis to check the suitability of merging the samples, unlike most studies [55].

It is also important to note two differences in how this paper has operationalized concept mapping, relative to standard practice. First, the authors noticed that nine items in the point map appeared unsuited to the grouping recommended by the hierarchical cluster analysis. Collectively they did not appear to form a natural conceptual grouping. The authors returned to the MDS results and noted a relatively low correspondence between the original data and the distance matrix for these items. This was supported by a (later) inspection of the raw sorting matrix for these items. Rather than follow the hierarchical cluster analysis results mechanistically, these items were not presented as belonging to any cluster and were instead considered *separately* at the interpretation stage. This treatment is consistent with Trochim’s advice that “the cluster analysis is viewed as suggestive and, in some cases, one may want to ‘visually adjust’ the clusters into more sensibly interpretable partitions of multidimensional space” [29] (p9).

Second, this concept mapping study chose to give a detailed presentation of the interpretation stage. The Scott & Ridings review found that most studies used only the research team to interpret the MDS findings, missing an opportunity for greater stakeholder engagement and associated validity gain [54]. Moreover, given the interpretive process involved, this paper presents a case for reporting the qualitative data collected during this stage (rather than simply the cluster labels created). Whilst the qualitative analysis was relatively simple, its use of narrative summaries and illustrative verbatim quotations gives readers a better understanding of how cluster labels were constructed and what underlies them.

## Conclusions

Attempts at quality improvement in community mental health services for older people are stymied by a lack of robust measures. Before designing a new instrument, researchers are encouraged to pay close attention to the construct of interest, and, for patient reported measures, to engage patient views in their development. Using concept mapping, this study identified seven key features of the patient experience in a sample of patients and practitioners from community mental health services for older people in England. These can be traced to enduring themes in the wider literature, and can be regarded as credible components for any assessment of the quality of patient experience. Subsequent stages of the research programme would use the results in the design and preliminary testing of a new questionnaire-based schedule.

## Additional files

Additional file 1: Stata Do File. Contains a STATA do file for multidimensional scaling, including the similarity matrix (raw data) used to construct the analysis presented within the manuscript. (TXT 46 kb)

Additional file 2: Dendrogram. Contains a dendrogram arising from the hierarchical cluster analysis described within the manuscript. (JPG 115 kb)

## Abbreviations

CMHT: Community Mental Health Team
MDS: Multidimensional Scaling
