# Supplementary figures and images for: The patient experience in community mental health services for older people: a concept mapping approach to support the development of a new quality measure
Source: BMC Health Serv Res. 2018 Jun 18;18:461. doi: 10.1186/s12913-018-3231-6 (PMC6006855; doi:10.1186/s12913-018-3231-6)

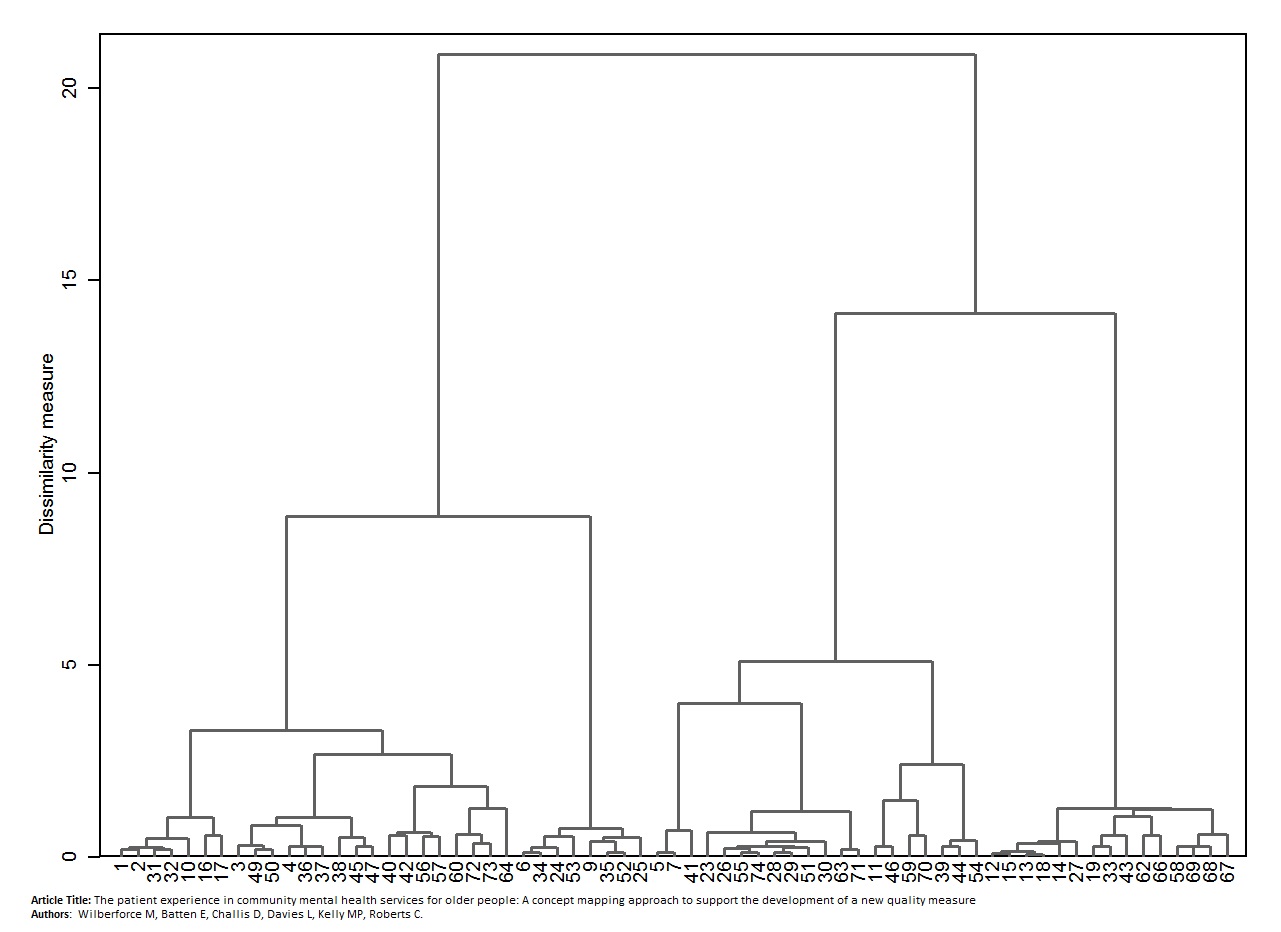

Supplement: Supplementary file 2 — Dendrogram. Contains a dendrogram arising from the hierarchical cluster analysis described within the manuscript. (JPG 115 kb) [file 12913_2018_3231_MOESM2_ESM.jpg]
